# The Role of Micro RNAs in Regulating PI3K/AKT Signaling Pathways in Glioblastoma

**DOI:** 10.30699/IJP.2022.539029.2726

**Published:** 2022-03-08

**Authors:** Roshanak Ghaffarian Zirak, Hurie Tajik, Jahanbakhsh Asadi, Pedram Hashemian, Hossein Javid

**Affiliations:** 1 Department of Medical Genetics and Molecular Medicine, Faculty of Medicine, Mashhad University of Medical Sciences, Mashhad, Iran; 2 Department of Medical Biotechnology, School of Advanced Technologies, Shahrekord University of Medical Science, Shahrekord, Iran; 3 Department of Clinical Biochemistry, Faculty of Medicine, Golestan University of Medical Sciences, Gorgan, Iran; 4 Jahad Daneshgahi Research Committee, Jahad Daneshgahi Institute, Mashhad, Iran; 5 Department of Medical Laboratory Sciences, Varastegan Institute for Medical Sciences, Mashhad, Iran; 6 Department of Clinical Biochemistry, Faculty of Medicine, Mashhad University of Medical Sciences, Mashhad, Iran

**Keywords:** Glioblastoma, Micro RNA, PI3K/AKT pathway

## Abstract

Glioblastoma is a type of brain cancer with aggressive and invasive nature. Such features result from increased proliferation and migration and also poor apoptosis of glioma cells leading to resistance to current treatments such as chemotherapy and radiotherapy. In recent studies, micro RNAs have been introduced as a novel target for treating glioblastoma via regulation of apoptotic signaling pathway, remarkably PI3K/AKT, which affect cellular functions and blockage or progression of the tumor. In this review, we focus on PI3K/AKT signaling pathway and other related apoptotic processes contributing to glioblastoma and investigate the role of micro RNAs interfering in apoptosis, invasion and proliferation of glioma through such apoptotic processes pathways. Databases NCBI, PubMed, and Web of Science were searched for published English articles using keywords such as 'miRNA OR microRNA', 'Glioblastoma', 'apoptotic pathways', 'PI3K and AKT', 'Caspase signaling Pathway' and 'Notch pathway'. Most articles were published from 7 May 2015 to 16 June 2020. This study focused on PI3K/AKT signaling pathway affecting glioma cells in separated subparts. Also, other related apoptotic pathways as the Caspase cycle and Notch have been also investigated. Nearly 40 miRNAs were found as tumor suppressors or onco-miRNA, and their targets, which regulated subcomponents participating in proliferation, invasion, and apoptosis of the tumoral cells. Our review reveals that miRNAs affect key molecules in signaling apoptotic pathways, partly PI3K/AKT, making them potential therapeutic targets to overcome the tumor. However, their utility as a novel treatment for glioblastoma requires further examination and investigation.

## Introduction

The most invasive and aggressive subtype of brain cancer in humans is glioblastoma, globally showing the highest incidence in Western Europe, Northern Ame-rica, and Australia; its prevalence in the United States has been more than 9 per 100,000 people (1-3). According to WHO classification, glioblastoma appears as grade I to IV; nevertheless, patients with different levels of glioblastoma have an average survival from 3 to only one year (4, 5). It is difficult to completely remove the tumor in surgery, and also there is a resis-tance to other treatments such as chemo- and radio-therapy (6, 7). This refers to some characteristics of glioblastomas: fast growth, high proliferation, the potential of self-renew, and absence of apoptosis (8), which may result from anti-apoptotic proteins overexpression (9). As recent statistics, the probability of patient survival with glioblastoma seems low. Thus, it is needed to find novel targets for quick diagnosis and efficient treatments (10).

Based on many studies on various cancers, including glioblastoma, changes in the expression level of some miRNAs could significantly alter tumor progression, and they contribute to biological processes of such cells as proliferation, differentiation, migration, and apoptosis (11, 12). These molecules are single-stranded RNAs with 18 to 24 nucleotides long being conserved highly (13, 14). They can bind to the 3′ untranslated region (UTR) of target genes and inhibit mRNA translation to regulate the protein expression related to a specific gene (15). As findings, increased or decreased levels of numerous miRNAs can induce apoptosis in tumor cells, suppressing their growth and inhibiting cancer development (12, 16).

Deregulation of the apoptotic pathway is the most crucial strategy of glioblastoma cells to fight current treatments (17). Hence, research into effective markers inducing apoptosis against cancer cells can be a novel treatment method. miRNAs participate in various pathways by targeting different genes and inducing oncogenic and anti-apoptotic effects on the function of glioblastoma (18). In this review, we summarize the main apoptotic pathways that affect glioblastoma and investigate the role of miRNAs in the mechanisms, including apoptosis.

## Material and Methods

**Search Strategy**

We conducted an electronic search for published articles in PubMed, NCBI, Scopus, and Google Scholar with no restrictions on publication date. The search of the literature was done independently by two first authors who applied no limitation in the language of the literature. The initial search was conducted using the following terms: ("micro RNAs" OR "miRNAs" OR "MiRNA") AND ("glioblastoma"), ("PI3K" AND "AKT"), in the title and/or abstract. 

**Inclusion and Exclusion Criteria**

**Fig. 1:**
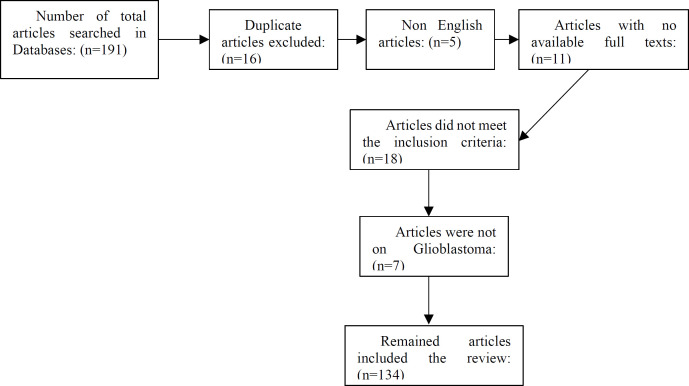
The flow diagram of excluding articles

Our search initially yielded 191 records in used databases that were managed in the EndNote X7.2.1 software. A total of 16 duplicate references were removed. After screening the remained articles, 5 of them were not in English, and 11 articles were not found in full text, so those were excluded. The full texts of the remaining 159 articles were carefully read. Then, 18 studies were excluded because there were no data on pathways of miRNA regulation. Additionally, another 7 case studies were not related to glioblastoma and those were also excluded. Figure 1 displays the detailed search results.

**1. AKT Signaling Pathway**

The AKT pathway is known to play an effective role in the biological functions of cells (19). In particular, it has been involved in tumorigenic active-ties in various cancers like glioblastoma (20, 21). AKT signaling pathway is a key link correlating with growth, proliferation, and invasion of tumor cells (22). It has been suggested that inhibition of AKT and its related signaling pathways can increase apoptosis in glioblastoma (23). Numerous miRNAs affect these pathways via direct and/or indirect targets resulting in suppressing glioblastoma or promoting oncogenic activities of the disease (24). 

1. **Correlation of AKT Pathway with Oncogenic EGFR and c-MET**

**Fig 2.**
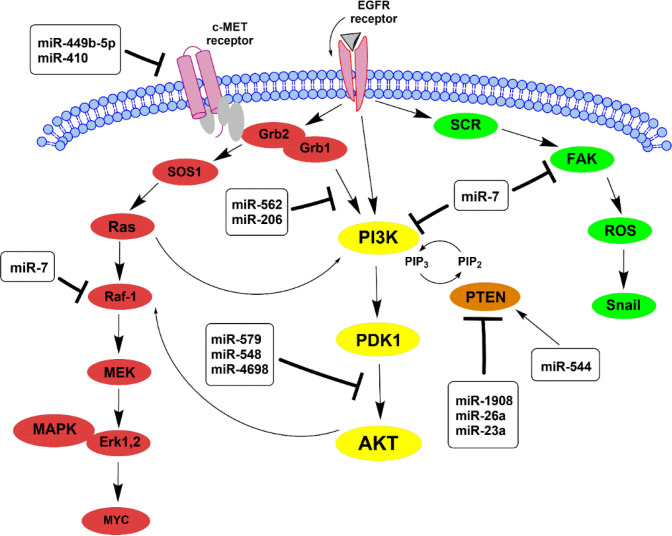
A schematic representation of PI3K/AKT, RAS/MAPK, and FAK/ROS signaling pathways participates in glioblastoma. They can activate by EGFR (Epidermal Growth Factor Receptor) and c-MET (Mesenchymal epithelial transition factor). The main pathway is PI3K/AKT, which activates other downstream glioma cells and affects apoptosis. The activation of PI3K results in phosphorylation of the PIP2 to generate PIP3. PIP3 activates PDK1 and, in turn, phosphorylates AKT. PTEN dephosphorylates PIP3 to PIP2 and acts as an antagonist of the PIP3 pathway. miRNAs indicating in frame affect their particular targets in these pathways, whether inhibition (showed by ┴) or inducing (showed by →).

According to results, overexpression of the Epidermal growth factor receptor (*EGFR*) gene is one of the most common changes that result in glioblas-toma (25). The *EGFR* has a crucial role in the up-regulation of PI3K/AKT/mTOR pathway, known as an effective downstream of EGFR, correlating with cell proliferation and tumor-forming (26). On the other hand, *EGFR* induces cell survival and invasion through other downstream pathways RAS/MEK/ERK (Figure 2) (27).

Some miRNAs can impact tumor activity by targeting such receptors and their downstream pathways; for example, miR-7 can suppress the expre-ssion of *EGFR* and inhibit the AKT pathway leading to a reduction in viability of gliomas (28). It has also been reported that miR-7 targets two EGFR downstream, PI3K and Raf-1, simultaneously suppressing both PI3K/ATK and Raf/MEK/ERK pathways (29). In addition, focal adhesion kinase (FAK) is identified as another target of miR-7 that negatively relates to this miRNA; miR-7 can reduce the invasion of glioblas-toma via directly targeting FAK (30). 

Furthermore, previous studies have reported that blockage of the c-MET expression can induce apoptosis in glioblastoma (31). The overexpression of the c-MET receptor results in tumor growth, and the c-MET/AKT signaling pathway can impact apoptosis and prolifer-ation in glioma cells (32, 33). As reported, miRNAs are closely correlated with c-MET, including miR-449b-5p and miR-410 that c-MET has been suggested as their direct target in glioblastoma (34, 35). Since the co-activation of c-MET and AKT can affect the apoptosis pathway in tumor cells, suppressing the c-MET/AKT pathway inhibits cell proliferation and promotes apoptosis by activating caspase3 downstream compo-nents (36, 37). Both tumor suppressors miR-206 and miR-562 regulate proliferation and induce apoptosis by inhibiting the c-MET/AKT signaling pathway (37, 38). 

**1.2. PI3K/PDK1/AKT Pathway and PTEN Regulation**

Activation of AKT is mainly correlated with PI3K and PTEN. PI3K is a kinase that phosphorylates PIP2 to produce PI3P, which in turn activates the AKT through direct binding to PDK1 (Figure 2) (39). Instead, PTEN, a tumor suppressor, applies reverse phosphorylation (PI3P is converted to PIP2), thus preventing AKT activation (40). As extensive evidence, there is frequent loss of PTEN in glioblastoma leading to a reduction in apoptosis (41, 42). Some miRNAs via PTEN regulation can affect growth, proliferation, and apoptosis in glioma cells; for example, oncomiR-26a and miR-1908 enhance the AKT pathway activity by down-regulation of PTEN level; besides, miR-23a as an oncogenic effector, targets PTEN to activate the PI3K/AKT pathway (43-45). A high level of miR-554 not only inhibits proliferation and invasion ability in gliomas but also increases cell apoptosis. miR-544 directly targets PARK7 protein to suppress its expression (46). PARK7 plays an important role in tumor development by binding to PTEN, and p53 leading to cell apoptosis inhibition (47, 48). 

**1.3. Oncogenic PI3K/AKT/mTOR Signaling Pathway**

As the results of previous studies, PI3K/AKT pathway is altered in 80% of glioma cells (49, 50). PI3K, a kinase controlling survival, proliferation, and apoptosis of the cells, can activate the AKT that in turn regulates its downstream targets as mTOR complex (39, 51-53). Mammalian target of rapamycin (mTOR) is a protein kinase including two complexes (mTOR1 and mTOR2) with different functions; either up- or down-regulation of some effectors in gliomas (54, 55). It has been suggested that mTOR2 is required for full activation of AKT and is known as the upstream kinase for it (56). The mTOR2 is formed via binding mTOR to RICTOR which is considered as an important oncogene in glioblastoma and its depletion leads to inactive mTOR2 complex (57, 58). Upregulation of AKT and overexpression of RICTOR in glioblastoma result in over-activation of mTOR2 that promotes proliferation and migration of tumor cells (55, 59). 

The increase in expression of some miRNAs can modulate PI3K/AKT pathway in gliomas; for instance, miR-579, miR-548, and miR-4698 inhibit proliferation and invasion of glioblastoma through affecting this pathway (60, 61). miR-6071 can bind to ULBP2 as the target gene in glioma cells and can inhibit the PI3K/AKT/mTOR pathway resulting in repress cell proliferation; thus, it promotes apoptosis (62). In addition, miR-489 and miR-326 via targeting SPIN1 and PKM2, respectively, can modulate the PI3K/AKT-/mTOR pathway to increase apoptosis and decrease invasion (63, 64). Interestingly, miR-652 plays an influential role in reducing tumor size in a patient with glioblastoma; miR-652 deactivates the AKT/mTOR pathway through its direct target FOXK1 (65).

**Fig. 3:**
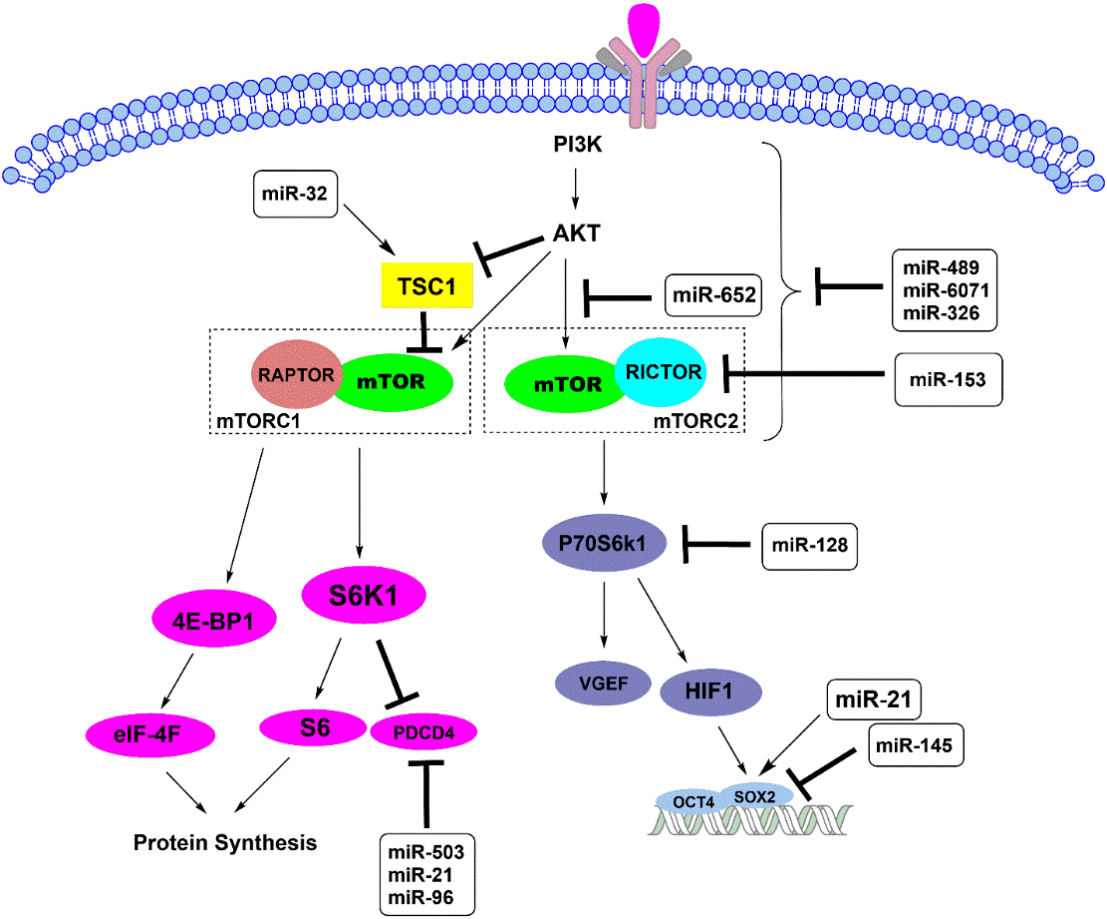
Schematic highlighting the mTOR complexes pathways and their downstream affect apoptosis in glioblastoma. TSC1 (Tuberous Sclerosis protein 1) and TSC2 can form a heterodimeric complex that inhibits the mTOR signaling pathway. RAPTOR and RICTOR are the main components of mTORC1 and mTORC2, respectively. The mTORC1 regulates protein synthesis by phosphorylating S6K1 and 4E-BP1, while mTOR2 affects gene expression, including SOX2, leading to cellular survival and tumor development

As a study, miR-153 is purposed as a tumor suppresser that affects RICTOR as the primary target. It has been suggested that the upregulation of miR-153 is negatively correlated with the downregulation of RICTOR and reduction of AKT activity (66). The results showed that miR-153 significantly inhibits cell growth and activates apoptosis in gliomas (66). Also, overex-pression of miR-128 has been reported to enhance apoptosis significantly in gliomas. Interestingly, miR-128 directly inhibits various targets, including mTOR, RICTOR, IGF1, and PIK3R1, which are members of mTOR signaling. Moreover, miR-128 targets P70S6K1, leading to repress the level of this protein and its downstream effectors, which are HIF-1 and VGEF (67). P70S6K1 protein is a tumorigenic downstream target of mTOR, which is activated through PI3K/PDK1/AKT pathway induced by insulin-like growth factor (IGF)-1 (Figure 3) (68).

Following mTOR signaling and P70S6K1 elevation, HIF-1 is activated, affecting Sox2; one of the two gene targets resulting in proliferation, migration, and tumorigenesis (Figure 3). It has been reported that upon overexpression of miR-145 as a tumor suppresser, Oct4 and Sox2 are targeted directly, leading to a decrease in the growth and migration of human glioma cells (68). In contrast, such a process is promoted via Sox2 activation by onco-miR-21 in gliomas (69).

Additionally, miR-21 exerts its oncogenic effects targeting other downstream of mTORC1 as S6K and 4E-BP1(70, 71). Activation of AKT leads to inhibit TSC1-TSC2 complex; thus, it provides conditions for binding of two components, mTOR and RAPTOR, that form the mTORC1 complex (72). Two downstream of mTORC1 interfere in cell growth and protein syn-thesis; S6K/S6 through blockage PDCD4 (program-med cell death) and 4E-BP1 via formation and activation of eIF-4F complex, can affect glioblastoma progression (73). The onco-miRNAs, including miR-21, miR-503, and miR-96 inhibit PDCD4 as the direct target that improves glioma viability and survival (70, 74, 75). On the other hand, miR-32 directly targets TSC1 and suppresses mTOR pathway leading to the reduction of angiogenesis levels (76).

**1.4. AKT Inhibits the Pro-apoptosis Downstream**

**Fig. 4:**
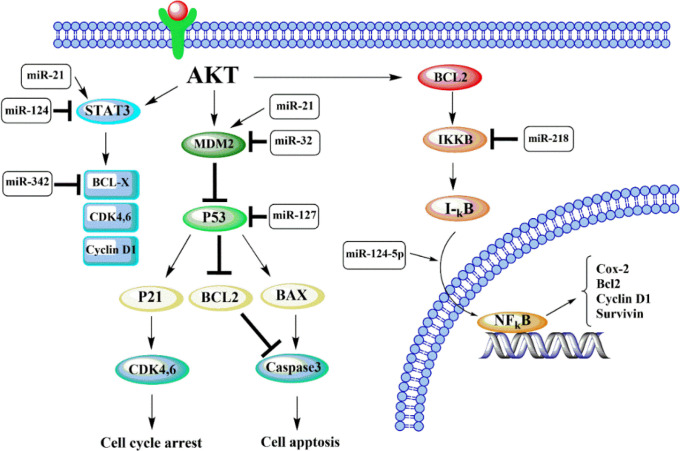
Schematic representation of apoptosis inhibition by AKT signaling pathway and its downstream effectors. Akt activates MDM2, which inhibits p53 via binding to this protein. P53 is a tumor suppressor that induces apoptosis by activating some downstream as p21 and BAX. Akt activation can trigger the IKKB, which decreases IκB as an inhibitor of NF-κB. IκB reduction allows NF-κB to move to the nucleus and activate the transcription of cell survival and apoptosis inhibitors, including the surviving and Bcl2 family

In glioblastoma, the AKT pathway is identified to promote tumor development due to the inhibition of apoptotic effectors and activation of anti-apoptotic components (77). Upon AKT activation, the down-stream, including GSK3, FOXO3, and BAD, are inhibited, resulting in blockage of the apoptotic pathways (Figure 4) (77, 78). Some miRNAs can affect the AKT signaling via regulation FOXO3 and its downstream effectors as Bim, p27, and cyclin D1; for instance, overexpression of miR-184 promotes the capacity of glioma proliferation by downregulating of FOXO3 (79). Also, as reported, the level of p27, an apoptotic effector, can be decreased by both miR-221 and miR-222 (80). These miRNAs suppress apoptosis by reducing P27kip-1 expression, which is known as a CDK (cyclin-dependent kinase) inhibitor (81). Additionally, according to the reports, oncomiR-10b prevents gliomas' death through targeting Homeobox D10 (HOXD10), pro-apoptotic gene, and Bim protein (82, 83). 

Furthermore, AKT activates anti-apoptotic components that play key roles in cellular functions (Figure 4) (77). As a result, STAT3 and β-catenin act as an oncogene in glioblastoma that can be modulated by various miRNAs (84, 85); miR-124 is suggested to suppress the tumor by inhibition STAT3 signaling pathway (86). In contrast, miR-21 can induce proliferation and invasion of gliomas through the STAT3/β-catenin pathway by targeting RECK (87). 

**1.5. AKT/NFkB/**Bcl2** Signaling Pathway**

One of the mechanisms of NFκB (Nuclear factor-kappa B) activation is the AKT phosphorylation of IκB mediated by IKKβ, a key regulator of NFκB (Figure 4) (88). Activation of such a pathway has an essential role in the capacity of tumor invasion, which is due to the up-regulation of matrix metalloproteinases (MMPs) (89). In contrast, miR-124-5p can target NRP-1 to promote tumorigenicity through PI3K/Akt/ NFkB pathway (90). Interestingly, some miRNAs regulate cellular functions and apoptosis through targeting the MMPs; miR-218 directly targets IKKβ that in turn reduces NFκB activity and MMP-9 expression in gliomas (91). Also, overexpression of miR-16 and miR-211 can suppress the MMP-9 and induce mitoch-ondrial apoptotic pathway by mediating caspase 3 and 9 (92, 93). However, miR-23a and miR-10b directly inhibit HOXD10 expression and induce gliomas invasion through modulating MMP-14 (44, 94).

The activated pathway of NFkB in glioblastoma can exert cell proliferation via inducing anti-apoptotic factors, including the Bcl2 family, Cyclin D1, and surviving (Figure 4) (95). Bcl2 family regulates apoptosis, including both pro- and anti-apoptotic components such as BCL-x and MCL1 (96). Both of these anti-apoptotic proteins can bind to BAK and BAX through the BH3 domain resulting in restraint of the apoptosis (97). In glioma cells, miR-342 targets directly BCL-x and MCL1, which significantly dec-reases their expression and induces apoptosis (98). In addition, miR-181 level in gliomas is inversely related to level of tumor, and Bcl2 is suggested as a target of this tumor suppressor (78). 

**1.6. AKT/MDM2 Signaling and p53 Regulation**

According to findings, in glioblastoma, phosphor-ylated AKT can enhance the expression of MDM2; a pro-oncogene downstream of AKT pathway (99). MDM2 is considered a key regulator of p53 protein levels via binding to p53 and promoting its degradation (100). The p53 is identified as a significant tumor suppressor that induces growth arrest and apoptosis via overexpression of its target gene as p21 and blockage of anti-apoptotic effectors as Bcl2 family (101). Increasing the level of p53 leads to upregulating downstream p21 that results in G_0_/G_1_ cell cycle arrest (102). It can also activate BAX/caspase 3 pathways leading to apoptosis in tumor cells (Figure 4) (103). Some miRNAs affect the components of such pathways to up- or down-regulate glioma functions; for instance, the expression level of p53 protein is reported to increase after the silencing of miR-127 (104) (Table 1). In addition, miR-32 can suppress tumor growth by direct targeting MDM2 and TSC1, two main p53 inhibitors (76). miR-21 targets various components of this pathway; it can increase the level of MDM2 by activating the AKT pathway leading to downregulation of p53 and apoptosis inhibition (105).

**Signaling Pathway Related to Caspase Family**

Caspase proteins, a family of proteolytic enzymes, play different roles in the cell; particularly, they are vital components of the apoptotic pathway (106). These proteins are classified into two categories; primary types, including caspase8 and caspase9, that activate the secondary caspases by cleaving, and conse-quently, activate caspases that cooperate in the apop-tosis process (107). According to the previous studies, the expression of caspase proteins has notably decre-ased in gliomas compared with normal cells associated with the reduction of apoptosis in glioblastoma (108).

Activation of some caspase proteins is effectively impressed with the receptors that are considered to initiate different cellular pathways; two of them, also known as the death receptors, are TNF-related apop-tosis inducing ligand receptor (TRAILR) and tumor necrosis factor receptor (TNFR) (109). The TRAIL receptor is involved in the activation of the caspase cascade in both extrinsic and intrinsic apoptosis pathways (110). It straightly activates the primary caspase8 via the extrinsic apoptotic path, which in subsequent, the downstream components, including caspase3 and caspase7, carry out the apoptosis process (111, 112). While intrinsic or mitochondrial pathway is tightly related to Bcl2 family including both anti- and pro-apoptotic proteins (113). Upon this pathway, a pro-apoptotic member of the Bcl2 family, protein Bid, is cleaved by activated caspase8 and then is translocated to mitochondria (112, 114). Consequently, some proteins such as cytochrome complex are released into the cytosol and combined with Apaf-1 resulting in Apaf-1/caspase 9 axis, which induces programmed cell death through activating caspases 3 and 7 (115). In glioma cells, some miRNAs can damage the signaling cascade of TRAIL and inhibit apoptosis; for instance, miR-21 can change TRAIL sensitivity by targeting the TAp63 (116). Likewise miR-30 targets the caspase3 and inhibits TRAIL-dependent apoptosis (116).

Furthermore, the TNF receptor triggers NFκB signaling cascade, which involves biological processes like cell survival and inflammation (117). Previous research has shown that the NFκB pathway intensifies because of the increased expression of the death rece-ptors in glioblastoma (118). According to evidence, the inappropriate activity of NFκB leads to inhibiting caspase8 and results in the resistance to apoptosis and induction of immortality in glioma cells (118).

Expression patterns of several miRNAs can alter the gliomas' functions by affecting the NFκB pathway; for example, the level of mir-218 expression is decreased in gliomas compared to normal tissues (91). Aberrant expression of this miRNA changes the transcriptional activity of NFκB by affecting the 3'UTR length of IKKβ (89). Another miRNA that is upreg-ulated in glioma cells is miR-21, which enhances the level of NFκB by targeting the *LRRFIP1 *gene (119). In addition, over-expression of mir-125b promotes the activity of NFκB by targeting both TNFAIP3 and NKIRAS2, leading to alter molecular mechanisms of main elements in the pathway (120). 

**Notch Signaling Pathway**

Many core functions in the cell are induced by notch signaling, such as differentiation leading to cell development, proliferation, and maintenance of stem cells, and it seems mutation of notch proteins have been related to developmental diseases; for example, schizo-phrenia may associate with mutation of notch4 (121, 122). Notch signaling prevents neural stem cells (NSC) from differentiation and provides the maintenance of NSCs in immature glia (123). Of note such function, it is considered that notch signaling may play a similar role in maintaining glioma stem cells, and it may interfere with tumorigenesis (121, 124). 

In glioblastoma, notch1 protein amplifies the transcription leading overexpress of epidermal growth factor receptor (EGFR) gene via TP53 signaling pathway that its mechanism is not clear enough (125). Following the block of Notch1 in gliomas, cell proli-feration decreases, and apoptosis increases. As the latter event, phosphorylation of AKT and STAT3 inhi-bits, while the pro-apoptotic form of caspase3 increases (126). Such pieces of evidence point out that the Notch1 pathway interferes in the growth and survival of glioblastoma cells (127). 

Several miRNAs have an effective role in up- or down-regulation of proliferation and apoptosis through notch signaling pathways in glioblastoma (128). The Notch1 3′-UTR sequence is involved in the luciferase receptor gene, which is significantly targeted by miR-146a, reducing its activity (129). It results in blockage of Notch1 expression and flows signaling pathway that reduces the proliferation of gliomas and induces apoptosis (8).

It has been reported that miR-34a regulates cell proliferation through various targets such as CCND1, CDK6, and the notch protein. In glioblastoma, TP53 is targeted by miR-34a, leading to its downregulation compared to normal brain cells. According to *in vivo* findings, increased expression of miR-34a can inten-sely inhibit glioma growth via targeting the c-MET and Notch signaling pathway (130). Thereby, miR-34a reduces glioma's proliferation and invasion, making it a tumor suppresser agent (131). Also, miR-145 can suppress the gliomas and induce apoptosis via a notch pathway but with a different target known as BNIP3 (132). In glioma cells, BNIP3 is in the nucleus that inhibits the apoptosis as an oncogene through both paths: blockage of TRAL and up-regulation of the notch signaling pathway (133). miR-145 can block BNIP3 expression by binding to it, which results in the reduction of some proteins, including notch1, Hes1, and P2, thus, the apoptosis increases in glioma cells (132). 

**Table 1:** Down- and up- regulated miRNAs and their functional pathways in glioblastoma

Tumor suppresser miRNAs			
Micro RNA	Target	Functional pathway	**Reference**
miR-34a	CDK6, CCND1, NOTCH	Notch signaling pathway	**(** 130 **, ** 13 **1)**
miR-145	BNIP3	Notch signaling pathway	**(** 133 **)**
miRNA146-a	Notch1	Notch signaling pathway	**(** 129 **)**
miR-7	PI3K, Raf-1, FAK	Raf/MEK/ERK pathway	**(** 29 **, ** 30 **)**
miR-449b-5p miR-410 miR-206 miR-562	c-MET	c-MET/AKT pathway	**(** 34 **, ** 35 **, ** 37 **, ** 38 **)**
miR-554	PARK7	PI3K/AKT pathway	**(** 46 **)**
miR-579 miR-548 miR-4698	-	PI3K/AKT pathway	**(** 60 **, ** 61 **)**
miR-6071	ULBP2	PI3K/AKT/mTOR	**(** 62 **)**
miR-489	SPIN1	PI3K/AKT/mTOR	**(** 63 **)**
miR-326	PKM2	PI3K/AKT/mTOR	**(** 64 **)**
miR-652	FOXK1	AKT/mTOR pathway	**(** 65 **)**
miR-153	RICTOR	AKT/mTOR pathway	**(** 66 **)**
miR-128	P70S6K1	AKT/mTOR pathway	**(** 67 **)**
miR-145	Oct4 and Sox2	AKT/mTOR pathway	**(** 68 **)**
miR-32	TSC1	AKT/mTOR pathway	**(** 76 **)**
miR-124	-	AKT/ STAT3 pathway	**(** 86 **)**
miR-16 miR-211	MMP-9	PI3K/Akt/ NFkB pathway	**(** 92 **, ** 93 **)**
miR-342	BCL-x , MCL1	AKT/NFkB/Bcl2	**(** 98 **)**
miR-181	Bcl2	AKT/NFkB/Bcl2	**(** 78 **)**
miR-32	MDM2, TSC1	AKT/ MDM2/p53	**(** 76 **)**
miR-218	IKKβ	PI3K/Akt/ NFkB pathway TRAIL/NFkB/ Caspase signaling cascade	**(** 91 **)**
**Onco-miRNA**			
Micro RNA	Target	Functional pathway	**Reference**
miR-26a miR-1908	-	PI3K/AKT pathway	**(** 43 **, ** 44 **)**
miR-23a	PTEN	PI3K/AKT pathway	**(** 45 **)** **(** 44 **)**
		AKT/NFkB pathway	
miR-21	Sox2, S6K, 4E-BP1 PDCD4 RECK	AKT/mTOR pathway STAT3/β-catenin pathway AKT/ MDM2 pathway	**(** 69 **, ** 70 **)** **(** 87 **)** **(** 105 **)**
	Tap63 *LRRFIP1*	Caspase signaling cascade TRAIL/ NFkB Caspase signaling cascade	**(** 116 **, ** 119 **)**
miR-503 miR-96	PDCD4	AKT/mTOR	**(** 74 **, ** 75 **)**
miR-30	Caspase3	Caspase signaling cascade	**(** 116 **)**
miR-184	-	AKT/FOXO3	**(** 79 **)**
miR-221 miR-222	P27kip-1	AKT/FOXO3	**(** 80 **)**
miR-10b	HomeoboxD10, Bim	AKT/FOXO3	**(** 82 **, ** 83 **)**
		AKT/NFkB	**(** 119 **)**
miR-124-5p	NRP-1	PI3K/Akt/ NFkB pathway	**(** 90 **)**
miR-127		AKT/ NFkB/p53	**(** 104 **)**
			
miR-125b	**TNFAIP3,NKIRAS2**	**TRAIL/NFkB Caspase signaling cascade**	**(1** 20 **)**

The table presents tumor suppressor and onco-miRNAs with observed effects upon their regulation in glioblastoma and the functional pathway impressing their direct targets.

## Conclusion

Apoptosis has been proved as a key function in all cells that occurs through various extrinsic and intrinsic cell pathways. Therefore, any dysfunction of up- or down-stream components can totally change the condition to benefit cell survival. Dysregulation in apoptotic signaling pathways is one of the most effective events confirmed as an indicator of cancer; as reported in the studies on glioblastoma, the signaling pathways alter to shut off apoptosis. Besides, there are numerous micro RNAs known as effective agents on the biological activity of tumor cells. 

In this review, we investigated the PI3K/AKT sig-naling pathways and related miRNAs affecting such pathways in glioblastoma. According to the collected evidence, the changes in the expression level of various miRNAs can notably affect the proliferation, invasion, and apoptosis in glioma cells. Many onco-miRNAs can upregulate gliomas' functions and help the cell survival, while others suppress the tumor growth and promote apoptosis. Overview of such effectors and their influence on the PI3K/AKT pathway sub-com-ponent can help us find novel procedures for gliob-lastoma treatment. However, it required: 1) to improve our understanding of their regulatory functions and their corresponding targets in other apoptotic path-ways, 2) to find some ways to control therapeutic responses and better management of glioblastoma .

## Conflict of Interest

The authors declared no conflict of interest.

## Funding

None.
